# Retinal and Choroidal Metastasis from a Lung Carcinoid Tumor

**DOI:** 10.18502/jovr.v20.17830

**Published:** 2025-12-19

**Authors:** Corrina P. Azarcon, Caroline M. Craven, Jill R. Wells

**Affiliations:** ^1^Department of Ophthalmology, Emory University School of Medicine, Atlanta, GA, USA; ^2^Department of Ophthalmology, University of Texas Health Science Center at San Antonio, San Antonio, TX, USA

**Figure 1:**
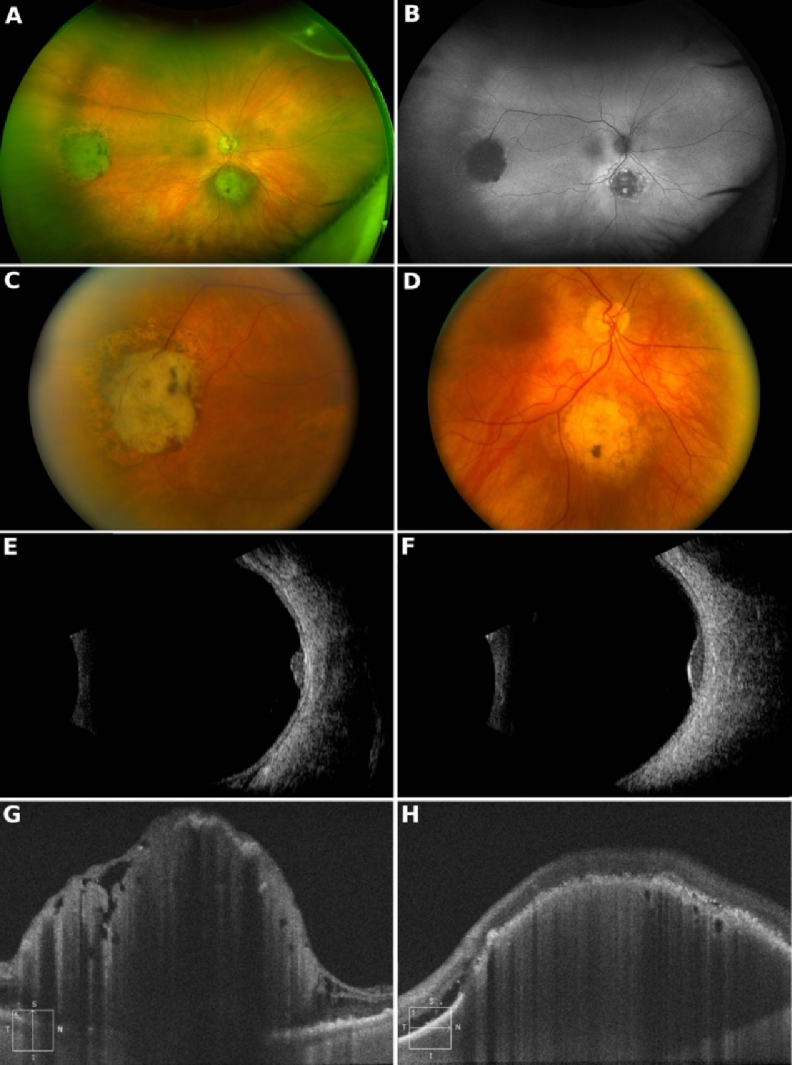
(A) Wide-angle fundus photograph of the right eye showing a retinal tumor in the temporal periphery and a choroidal tumor inferior to the disc. (B) Fundus autofluorescence photograph of the same eye. (C) Color photo of the retinal tumor. (D) Color photo of the choroidal tumor. (E) B-scan over the retinal tumor. (F) B-scan over the choroidal tumor. (G) Optical coherence tomography (OCT) scan over the retinal tumor. (H) OCT scan over the choroidal tumor.

##  PRESENTATION

Carcinoid tumors are low-grade malignancies that typically arise from the gastrointestinal and respiratory tracts. Although these tumors are indolent and associated with relatively longer survival rates, metastasis occurs in 5–71% of patients.^[1]^ While involvement of the choroid in hematogenous spread of malignancy is not uncommon owing to its prominent vasculature,^[2]^ retinal metastasis is exceedingly rare, accounting for < 1% of intraocular metastases.^[3]^ Only two case reports have described retinal metastasis from a carcinoid tumor, with imaging modalities limited to somatostatin scintigraphy^[4]^ and computerized tomography (CT) scans.^[5]^ This report presents the first detailed description of multimodal imaging features of concurrent choroidal and retinal lesions in a patient with metastatic carcinoid tumor.

##  DISCUSSION

A woman in her 70s with a lung carcinoid tumor, who had previously been treated for choroidal metastasis in both eyes, was referred for ophthalmic evaluation in the context of extensive metastatic disease. She had been diagnosed with the primary lung carcinoid tumor 15 years prior and had undergone multiple chemotherapeutic treatments and local interventions. She was treated with photodynamic therapy in the right eye for a choroidal mass inferior to the optic nerve and plaque radiotherapy for a macular choroidal mass in the left eye. Both tumors regressed following treatment.

At the time of her presentation, she had a visual acuity of 20/25 in the right eye and hand motions in the left. Fundoscopic examination of the right eye revealed recurrence of the choroidal mass inferior to the right optic nerve, and a new retinal lesion in the temporal periphery. The left eye showed a regressed, atrophic choroidal mass in the macula. Multimodal imaging was employed to further characterize the tumors in the right eye.

Wide-angle fundus photography demonstrated a retinal tumor located in the temporal periphery and a choroidal tumor situated inferior to the optic disc in the right eye [Figure 1A]. Fundus autofluorescence imaging showed blocked autofluorescence corresponding to the retinal tumor and patchy hypoautofluorescence associated with the choroidal tumor [Figure 1B]. On color fundus photography, the retinal tumor appeared as a superficial yellow mass with prominent vasculature and surrounding pigmentary changes [Figure 1C], while the choroidal tumor presented as a deep yellow mass with occasional areas of pigmentation [Figure 1D]. B-scan ultrasonography of the right temporal retina showed a solid retinal mass with sharply elevated borders, while the choroidal tumor exhibited a more gradual elevation [Figure 1F]. Optical coherence tomography (OCT) of the temporal retinal lesion revealed an irregular retinal tumor confined between an intact internal limiting membrane and the retinal pigment epithelium-Bruch's membrane complex [Figure 1G]. OCT of the choroidal mass showed a dome-shaped choroidal lesion with overlying subretinal fluid [Figure 1H].

Carcinoid tumors, due to their low-grade nature, are associated with longer survival rates in comparison to other metastatic malignancies.^[1]^ However, retinal metastasis from various carcinomas and cutaneous melanoma is uniformly linked to a poor prognosis and typically indicates terminal-stage disease with widespread tumor dissemination.^[3]^ In our case, the development of a retinal tumor coincided with a rapid decline in the patient's overall health, leading to her death.

##  Financial Support and Sponsorship

None.

##  Conflicts of Interest

None.
